# Cancer and Traditional Medicine: An Integrative Approach

**DOI:** 10.3390/ph18050644

**Published:** 2025-04-28

**Authors:** Cheruthazhakkat Sulaiman, Blassan P. George, Indira Balachandran, Heidi Abrahamse

**Affiliations:** 1 Phytochemistry Division, Centre for Medicinal Plants Research, Arya Vaidya Sala, Kottakkal 676503, Kerala, India; sulaimanct@aryavaidyasala.com (C.S.); indirapa@aryavaidyasala.com (I.B.); 2 Laser Research Centre, Faculty of Health Sciences, University of Johannesburg, P.O. Box 17011, Doornfontein 2028, South Africa; habrahamse@uj.ac.za

**Keywords:** cancer, medicinal plants, phytochemicals, natural products, traditional medicine

## Abstract

Despite numerous advances in treatment, cancer still remains a leading cause of death worldwide. Given the significant health and economic burden this disease imposes, it is important to explore more effective treatment strategies. A major drawback of conventional cancer therapies is the persistence of drug resistance, adverse reactions to chemotherapy, digestive damage, reduced quality of life, and high treatment costs. To address these challenges, researchers have been investigating the utility of using complementary and alternative medicine (CAM) alongside conventional cancer treatments. Some of these CAM approaches have been reported to enhance patients’ quality of life and reduce the severity of adverse effects from conventional therapies. This review explores the utility of traditional Chinese, Korean, Indian, Japanese, and South African medicines as supportive therapies for conventional cancer treatments. We also discuss the concept of integrative oncology and its global relevance, as CAM approaches typically emphasize whole-person care, including diet, lifestyle, and mental/emotional well-being. In addition, we identify key active phytochemicals and herbal medicines used in these traditional systems for cancer treatment. Our discussion aims to provide a foundation for future research into integrative oncology, fostering an interdisciplinary and complementary approach to cancer management.

## 1. Introduction

Traditional herbal medicines and medicinal plants have a significant role in new drug development, particularly for cancer and other infectious diseases. Phytochemicals with diverse chemical structures and various therapeutic properties are the major ingredients of many newly developed drugs. Research on natural products and herbal drugs explains their significance for infectious diseases and cancer [1]. Natural products, especially herbal medicines, have been studied for their healing efficacy against various human ailments, and it led to the investigation and separation of a variety of phytochemicals with proven biological properties. In the case of cancer therapy, around 60% of anticancer drugs are originated from either natural products or their derivatives [2]. Various standardized herbal extracts are also being used for the management of and improvement in the quality of life of cancer patients.

Cancer is still a global public health concern and the second most common cause of death following cardiovascular diseases. The diagnosis and treatment of cancer were adversely affected during the COVID-19 pandemic, and reduced access to cancer care centers because of the pandemic situation might have affected the increased mortality rates of cancer during the past three years [3]. Various research activities related to diagnostic measures, treatment protocols, and prophylactic management have been developed considerably using new modalities and technologies. Additionally, novel approaches and promising new drugs have been developed and utilized for cancer treatment [4].

The phytochemicals isolated from various medicinal plants are well known to possess considerable anticancer properties that are acted on through various mechanisms that can alter proliferation, migration, and apoptosis in cancer cells. There is a number of phytoconstituents that are known to be principal sources of many chemotherapeutic drugs [5]. However, developing active phytochemicals into drugs has remained challenging due to difficulties in the commercial-scale production, purification, and stability of the developed drugs. Nowadays, pharmaceutical companies have reduced their efforts in natural product research for drug development and primarily rely on synthetic compounds or biologics [6].

Medicinal plants are the principal ingredients used in various traditional systems of medicine, like Traditional Chinese Medicine (TCM), Ayurveda etc. Herbal medicine is one of the oldest forms of therapy. In all parts of the world, independent forms of healing with plants have developed over the centuries, such as Ayurveda in India, Kampo medicine in Japan, Sa-sang in Korea, TCM in China, etc. [7,8]. Recently, the use of herbal extracts with proven therapeutic activity as a supportive medicine is more popular as part of an integrative approach to cancer treatment. Many of the medicinal plant extracts with therapeutically active phytoconstituents can act as an adjuvant for improving the quality of life and extending the life span of cancer patients.

The drug development process from plant extracts is a tedious process with enormous cost involvement. The concern regarding the toxic effects of isolated compounds is a major apprehension in the drug development process. Herbal drugs and plant extracts are considered safer compared to synthetic chemicals [9]. Hence, an in-depth toxicity investigation is essential before evaluating its therapeutic properties. Generally, medicinal plant extracts are safer than single molecules isolated from plants. Considering the example of reserpine, the antihypertensive drug commonly employed in the treatment of high blood pressure, which is isolated from *Rauvolfia serpentina* and is being used in Ayurveda for safe and effective treatment of hypertension, was banned due to its adverse effect; however, *Rauvolfia serpentina* is still in use in Ayurveda without any toxic side effects [10,11]. The toxicological profiles of isolated single compounds and plant extracts are significantly different in terms of clinical effects.

Medicinal plants contain several metabolites and active phytochemicals, such as polyphenols, terpenoids, alkaloids, flavonoids, flavanones, saponins, etc., which are useful for cancer chemoprevention and treatment [12]. Several medicinal plant extracts can inhibit the progression and development of cancer [13]. Herbal extracts are reported to contain active phytochemicals with anticancer potential [14,15,16,17,18,19,20]. This review highlights the scientific and technical improvement of various traditional medicines that researchers hope can provide relief to cancer patients as an integrated medicine along with conventional cancer therapy, mainly focusing on improving quality of life. In addition, the review also focuses on the global status of integrative oncology, integrative clinical oncology, the advantages of herbal drugs, and clinical studies conducted using herbal medicine.

## 2. Traditional Medicines and Cancer Care

During the last few decades, several complementary and alternative medicines have been utilized for the management of various types of cancer, including Traditional Chinese Medicine (TCM) and Ayurveda. The role of genetic and epigenetic changes in the initiation and progression of cancer has been evidenced by recent studies, which have strengthened the understanding of the involvement of traditional systems of medicines like TCM and Ayurveda in cancer management [21]. TCM has a long history of cancer treatment, which has been evidenced by the classical literature of Chinese medicine. Numerous studies have shown that combining TCM with other conventional cancer treatment can enhance the survival rates of cancer patients, improve their quality of life, and stop the growth and spread of cancerous cells. Huangqin is an important form of TCM that is being used for the treatment of cancer [22,23]. The diagnosis and treatment of tumors were discussed in ancient TCM texts like Yellow Emperor’s Inner Canon more than 2000 years ago. The major concepts of TCM are regulating body immunity, eliminating pathogens, and treating both the symptoms and the root cause of the disease. TCM has been reported to enhance the effectiveness of radio- and chemotherapy by strengthening the spleen and stomach, nourishing the liver and kidneys, and eliminating toxins from the body. TCM combined with chemotherapy has shown promising results in improving efficiency, with fewer adverse side effects [24]. During the last several decades, it has been shown that TCM is effective in cancer therapy, demonstrating minimal toxicity and improved efficacy as an adjuvant to radio- and chemotherapy [25,26,27].

Ayurveda is India’s ancient health care system, originating in Vedic times around 5000 years ago, and is one of the oldest traditional medical systems with a holistic approach to health, dealing with various aspects such as prevention, diagnosis, and treatment through detailed personalized regulation of food, nutrition, and diet as per the individual constitution, or “Prakriti”. The scientific basis of Ayurvedic principles, such as Prakriti, has been correlated with human biology and genomics [28,29,30]. In Ayurveda, cancer is defined as inflammatory or non-inflammatory swelling and mentioned either as “Granthi” (minor neoplasm) or “Arbuda” (major neoplasm). There are three important and basic factors involved in the normal functioning of the body according to Ayurveda, such as the nervous (“Vata”, or air), venous (“Pitta”, or fire), and arterial systems (“Kapha”, or water). According to the Ayurvedic system, in the case of malignant/cancerous tumors, these three basic systems are out of control (“Tridoshas”). These factors lose mutual coordination, which leads to tissue destruction, resulting in critical conditions. Tridoshas cause excessive metabolic crises, resulting in proliferation and tumorigenesis [31].

The “shared pathology” concept among cancer and other metabolic syndrome underlines the Ayurvedic perception of cancer. Studies have shown that gene signatures/expression patterns are strongly associated with cancer, inflammatory diseases, cardiovascular conditions, and gastrointestinal disorders. Most of the genes common to cancer and metabolic diseases regulate cholesterol biosynthesis and lipid metabolism [32]. Chronic inflammation is one of the major pathways common to both of these diseases and is considered a significant hallmark of tumorigenesis. Studies have shown that epigenetic dysregulation, diet, environmental factors, and immune function significantly affect the phenotype of a cancer patient. Ayurveda also considers epigenetic factors, diet, and environmental features as vital regulators in the case of a disease like cancer. In Ayurveda, the concept of “shared pathology” between cancer and metabolic syndrome has been correlated with interactions between vitiated Doshas and weak tissues (Dhatus), leading to systemic malfunctions that can manifest as cancers of particular organs [33,34,35].

**Figure 1:**
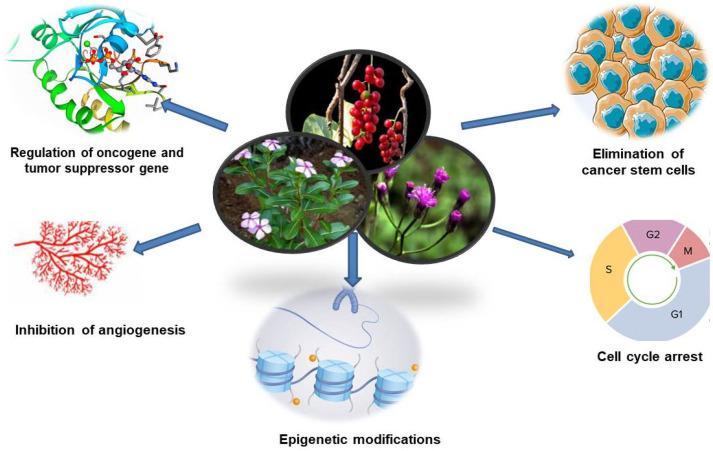
Schematic diagram demonstrating the application of herbal drugs in cancer therapies.

The therapeutic efficacy of an Ayurvedic herbal formulation depends on the multiple phytoconstituents extracted from the raw plant drugs. Bioactive compounds are reported to act as epigenetic regulators that modify gene expression. Various phytoconstituents of Ayurvedic medicinal plants might be capable of reversing epigenetic aberrations during carcinogenesis. The reversal of epigenetic aberration might be helpful in cancer treatment without adverse side effects. Many Ayurvedic formulations have been reported to contain numerous phytochemicals and exhibit significant anticancer activity (Figure 1) [36,37,38].

Traditional Korean Medicine (TKM) has been in practice to treat various ailments for many years. The Joseon period (1392–1910) saw the creation of the first encyclopedia of Oriental medicine, which summarized Korean Oriental medicine. Later, Sasang Constitutional Medicine (SCM) was established based on the principles of Korean medical tradition. TKM classifies humans into four unique constitution types: Taeyangyin (TY type), Tae-eumin (TE type), Soyangyin (SY type), and Soeumin (SE type), depending on the nature of an individual’s physiological, psychological, and physical characteristics, which lead to differential responses to herbs [39,40]. Similar to the Ayurvedic concept, TKM also emphasizes holistic modulation and improvement of the whole body rather than just eliminating the cancer cells. The patient, rather than just the tumor mass, is considered as a whole to treat the various illnesses. Chronic diseases, including cancer, are considered to be caused by a continuous imbalance of body, mind, and spirit. The holistic approach of TKM addresses the broad spectrum of diagnosis, treatment, and prognosis and suggests lifestyle modification for a person diagnosed with cancer. Lifestyle and diet modification are common approaches of various traditional medicine systems in the treatment of chronic diseases like cancer [41].

Traditional Japanese medicine, popularly known as Kampo medicine, was originally developed from ancient Chinese medicine and established during the 16th and 19th centuries in Japan. Kampo medicines can act as polypharmacological therapy, and hence each Kampo formula can address multiple symptoms. Supportive cancer care was officially incorporated into treatment schedules in Japan following the establishment of the Japanese Association of Supportive Care in Cancer (JASCC) in 2015. Kampo has been included in the JASCC for various diseases, including cancer care, and has been reported to play significant roles in preventing the adverse side effects of anticancer agents [42].

**Figure 2:**
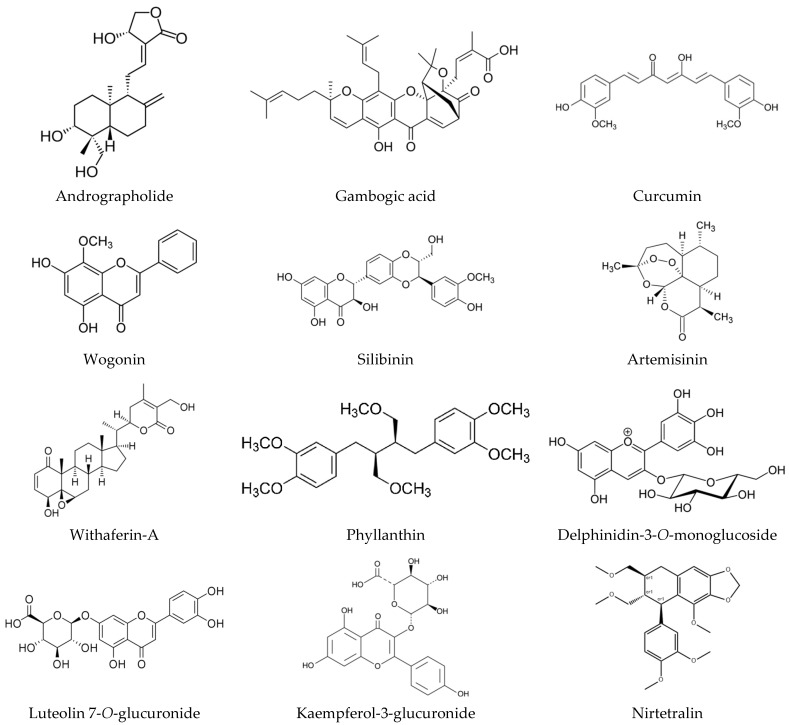
Chemical structures of some important anticancer compounds present in various traditional systems of medicine.

Other traditional medicines, like Unani, Siddha, traditional Persian medicine, etc., also emphasize holistic approaches towards cancer management. Cancer, known as “Sartān” in Unani medicine, is defined as “Saudawi warm” (melanotic swelling), which occurs due to the combustion of either “Safra” (yellow bile) or both “Balgham” (phlegm) and “Safra” (yellow bile) in the body. Various medicines based on plant, animal, and mineral origins along with diet therapy are the treatments recommended in Unani [43,44,45]. Anticancer activities of some Siddha medicines have also been reported earlier [46]. Important medicinal plants and formulations used in various systems of traditional medicine for the management of cancer are presented in Table 1, and Figure 2 presents the chemical structures of some important anticancer compounds present in various traditional system of medicines.

## 3. Advantages of Herbal Drugs

Research on the development of the medicinal benefits of plants is in increased demand in both developing and developed countries. In many African countries, including South Africa, traditional herbal medicine is being used for the treatment of several chronic diseases [71,72,73,74]. Herbal medicines are plant-derived materials with minimal cost and processing and have been used to treat various illnesses. They have been used for many years by local healers or for traditional healing practices. Herbal drugs or formulations have been mainly used for health promotion and disease prevention. However, their use has increased due to the side effects of modern medicine, such as fatigue, diarrhea, constipation, insomnia, vomiting, anemia, hair loss, variations in blood pressure and sugar, etc. The poor therapeutic outcomes and restricted treatment options for various serious diseases, lack of efficiency of common treatments, and major side effects or risks related to conventional medicine promote the use of better or safer herbal and natural medicines [75].

African traditional medicine using medicinal plants is continuing to play a significant part in the holistic management of wellbeing through preventive, therapeutic, and palliative care. Medicinal plant research is based on traditional ethnobotanical knowledge and is supported by investigative laboratory procedures, such as the use of different polar solvents for the extraction of medicinal plant materials, qualitative and quantitative phytochemical analysis, activity-guided fractionation, isolation of bioactive compounds, and structural characterization of the isolated active compounds. The crude extracts and isolated bioactive compounds of interest are finally tested for various pharmacological properties using in vitro and in vivo models to confirm their biological activities [76].

The revolution of medicinal plants/herbs and their cell death induction mechanism action would support an alternate efficient therapy and cancer prevention. For decades, several drug lead compounds have been derived from potential bioactive compounds found in plants. Anticancer drug discovery from medicinal plants started in the late 1960s with the discovery of podophyllotoxin, followed by the discovery of vinca alkaloids, campthothecin, Taxol, etc. More than 100 species of medicinal plants exhibit anticancer properties. Most of these bioactive compounds exhibit more than one pharmacological property and are applicable in various cancer therapies. For example, Taxol has been found to be beneficial in the treatment of ovary, breast, and other cancer types, and podophyllotoxin’s structural analog molecule etoposide is effective in the treatment of lung and testis cancers [77,78,79,80,81].

Several clinical studies/trials have reported that the use of herbal medicines in cancer patients minimizes the mortality rates and increases the survival and quality of patients’ life by lowering the symptoms and adverse side effects of conventional drugs. Reports also show that medicinal herbs demonstrate chemopreventive action against some cancer types [82,83,84,85]. Preclinical studies have constantly shown that several herbal medicines have antiapoptotic, anti-inflammatory, cell-regenerative, and antioxidant effects on several cancer cells. Nevertheless, the clinical evidence pertaining to the efficiency of many herbal medicines used in cancer is largely inconclusive [86,87]. The report also showed that African and Asian countries had the highest prevalence of herbal medicine use for cancer, while Oceania had the lowest. Likewise, many more cancer patients from low- and middle-income countries used herbal medicine than those from high-income countries. The difference in prevalence across these countries is based on the variations in geographical characteristics, such as the conditions of easy availability of herbs, cultural beliefs, and low regulation of herbal medicines [88,89,90,91,92]. The low levels of income in low- and middle-income countries could explain the higher usage of herbal medicines, as cancer patients may be unable to afford conventional cancer treatments due to financial constraints. Moreover, in some countries, deep cultural practices also make them adopt herbal medicines. Studies show that in many Asian countries, regardless of availability and budget to receive high-quality cancer therapies, patients continue to use herbal medicine along with conventional therapies.

Several Chinese herbal medicines are used to enhance cancer treatments and minimize adverse side effects in combination with conventional therapies. Many clinical studies have reported the beneficial effects of herbal medicines as alternative and complementary cancer treatment and have reported that they improve the quality of life in cancer patients when used in combination with existing conventional therapies [93]. Herbal medicines fight cancer in a different way than conventional drugs; they strengthen the immune system by preventing cancer cell proliferation, inhibiting angiogenesis, and detoxifying the body by scavenging free radicals that cause mutations that lead to carcinogenesis [94] and create an adverse environment for cancer cell proliferation [95]. A study in the UK reported that 25% of cancer patients consulted herbal medicine practitioners, though this number is likely underestimated [96]. Similar to this report, in Canada, a study reported that 20% of breast cancer patients use herbal medicines [97], whereas in the United States, this percentage is above 65% [98], and the rates are significantly higher than those in other reports on the general population or among other cancer groups [99].

## 4. Integrative Clinical Oncology

**Figure 3:**
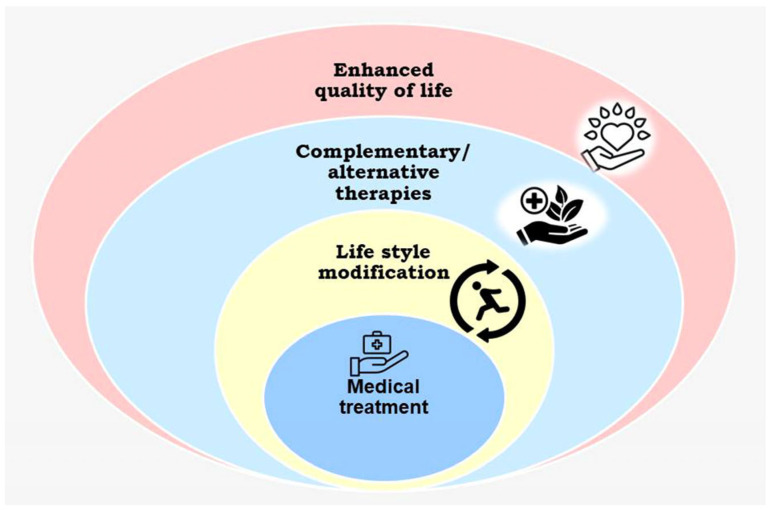
Schematic diagram of an integrative oncology approach.

Various literature surveys revealed that the use of alternative and complementary medicines enhanced the prognosis of cancer patients and played a significant role in improving quality of life of advanced-stage cancer patients. Providing alternative care along with conventional cancer treatment will be beneficial to patients suffering adverse effects of chemo- and radiotherapies. Integrative oncology in a broad sense is patient-centered, evidence-based, comprehensive cancer care that uses mind–body practices, herbal formulations, and lifestyle modifications from different traditions in conjunction with conventional cancer treatments. It prioritizes safety and the best available evidence to offer appropriate therapeutic interventions along with conventional care (Figure 3).

A combination of clinical procedures and complementary therapies has been reported successfully in certain cancers. The management of pancreatic ductal adenocarcinoma (PDAC) with combined clinical and complementary measures has been previously reported to enhance both the quality of life and the survival time of patients. Authors have also reported that several opportunities to be investigated in the future include emerging modalities, precision medicine, the nerve input to tumors, and, significantly, clinical trials [100]. Another study in PDAC groups with alternative therapies including dietary factors also showed positive results. Evidence-based complementary therapies in cancer treatment have demonstrated hopeful results in the clinical setting and have gained attention from oncologists worldwide. It has been concluded that overall integrated management of PDAC is currently likely to produce the best outcome for patients, and a wide range of complementary measures is available for this purpose [101].

The health concepts of various complementary medicines are unique in their diagnostic and therapeutic aspects. There are many scientific studies regarding the anticancer potential of herbal formulations that can be used as an adjuvant to chemo-/radiotherapy in controlling the adverse side effects and improving the patient’s quality of life [102,103]. Siddha, another traditional system of medicine practiced in the southern part of India, considers malignant tumors “Putru”. The materia medica of Ayurveda and Siddha are similar in that both depend on medicinal plants; however, most Siddha drugs are of metal and mineral origin along with herbs. Various herbo-mineral combinations are used for the management of cancer in Siddha [104].

## 5. Global Status of Integrative Oncology

The massive number of cancer cases and mortality rates is challenging current cancer care worldwide, particularly in low- and middle-income countries. Integrating conventional treatment modalities with traditional, complementary, and integrative medicines may reduce the burden due to the huge expenses of cancer treatment. Many alternative therapies, like lifestyle modifications, mind and body therapies, and the use of natural products, are viable approaches for improving symptom management and quality of life in cancer care. Integrative cancer management is widely used by patients in Latin American countries, and it is estimated that between 50% and 90% of adult or pediatric patients with cancer use TCM [105,106]. In China, approximately 75% to 80% of patients use TCM after cancer diagnosis. TCM is widely integrated in oncology departments within hospitals in China in association with conventional cancer treatments [107,108]. The holistic approach to treatment in various Indian traditional systems of medicine offers pluralistic health care for cancer patients. Ayurveda, an ancient Indian system of medicine, has been in practice for several thousand years, with its holistic approach to the individual and personalized treatment approaches. Several herbal formulations used in Ayurveda have been reported to improve the quality of life in cancer patients [109]. Integrative oncology can make a significant contribution to global cancer care. Programs must be initiated with evidence-based approaches for the successful integration of conventional cancer care with complementary and alternative systems across the world.

## 6. Status of Integrative Oncology in South Africa

In South Africa, even though more than 27 million people rely on traditional medicine for various healthcare needs, the use of medicinal plants for cancer care remains under-researched. A limited number of ethnobotanical studies have been conducted on cancer therapies. Ethnobotanical studies have been performed in KwaZulu-Natal, the Eastern Cape, and the Western Cape provinces [110,111]. Another ethnobotanical study reported on the use of medicinal plants for reproductive diseases in the Capricorn, Sekhukhune, and Waterberg districts of Limpopo Province. In this study, breast cancer was grouped as a reproductive disease [112]. Lead candidate molecules, such as sutherlandiosides, sutherlandins, hypoxoside, and pittoviridoside, were identified for toxicity studies and clinical trials [113]. In the Eastern Cape Province, being one of the poorest provinces in South Africa, the people heavily rely on medicinal plants for their primary healthcare, including for cancer, due to the cost-effectiveness, cultural beliefs, and perceived safety. This province is predominantly inhabited by the isiXhosa-speaking people of Cape Nguni ancestry, and the use of medicinal plants for treating diseases is an essential part of their cultural life. An ethnobotanical survey conducted in this project revealed that more than twenty-four different plant species have been explored for their anticancer properties. *Aspalathus linearis*, *Agapanthus africanus*, *Cannabis sativa*, *Catharanthus roseus*, *Eucomis autumnalis*, *Euphorbia ingens*, *Hypoxis argentea*, *Pittosporum viridiflorum*, *Solanum aculeastrum*, *Sutherlandia frutescens*, etc., are some of the important anticancer plants reported in South Africa [114,115].

## 7. Anti-Inflammatory and Antioxidant Activities of Traditional Medicines

Anticancer properties of many traditional medicines are highly correlated with their anti-inflammatory and antioxidant properties. Several compounds derived from Ayurvedic and TCM plants are potential anticancer agents and are also used as dietary supplements [116,117]. Ayurvedic medicinal plants such as *Boswellia serrata*, *Commiphora wightii*, *Hemidesmus indicus*, *Aloe vera*, *Withania somnifera*, *Zingiber officinale*, *Berberis aristata Shorea robusta*, *Curcuma longa*, *Punica granatum*, and *Psidium guajava* are well known for their anti-inflammatory and antioxidant properties [118]. Herbs used in TCM and their active compounds have been shown in many in vitro and in vivo models to inhibit inflammatory responses in different organs, including the lungs, esophagus, brain, colon, skin, prostate, mammary glands, liver, and pancreas [119]. In South Africa, more than 100 plant species of 60 families are used to treat pain-related inflammatory disorders. The major phytochemicals of these plants are phenolics, saponins, terpenoids and alkaloids. The therapeutic properties attributed to these plant metabolites are highly correlated with their antioxidant, anti-inflammatory, and anticancer activities [120].

Ye Ju Hua and Ju Hua have a long history of use in traditional Chinese and Korean medicine for the treatment of inflammation, hypertension, and respiratory diseases. Ojayeonjonghwan, an important traditional Korean medicine, has been reported to significantly decrease LPS-stimulated secretions and mRNA expressions of tumor necrosis factor (TNF)-α, interleukin (IL)-6, and IL-1β, and increase inhibition rates of TNF-α, IL-6, and IL-1β [121]. Kampo medicines such as Hangeshashinto (HST), Orengedokuto (OGT), Inchinkoto (ICT), Orento (ORT), Byakkokaninjinto (BKN), Juzentaihoto (JTT), Hochuekkito (HET), and Shosaikoto (SST) were being used for inflammatory diseases [122]. Antioxidants and anti-inflammatory agents have potential therapeutic value in cancer management based on mitigating oxidative stress and inflammation, both of which are implicated in cancer initiation and progression. Antioxidants function by scavenging reactive oxygen species (ROS), which can induce DNA damage and mutations, thereby contributing to carcinogenesis. Meanwhile, anti-inflammatory compounds can modulate immune responses and attenuate chronic inflammation, a condition that may facilitate tumor growth, metastasis, and angiogenesis [123,124].

## 8. Disadvantages of Traditional Medicines

Quality and safety are major concerns in traditional medicines. Ensuring the consistent quality and standardization of herbal products is crucial to guarantee their safety, efficacy, and reproducibility. An important problem the alternative medicine industry faces today is the non-availability of genuine herbs in required quantities. This leads to adulteration and unauthorized substitution, which affects the quality of medicines adversely. Ensuring quality, safety, and consistency of herbal medicine is a critical need for the industry. Various levels of standardization of raw drugs used in traditional medicines, including pharmacognostic, physicochemical, phytochemical, and molecular evaluation, are required for developing standardized quality control profiles of traditional medicine. By implementing strict testing and quality standardization protocols, potential risks such as contamination, adulteration, and the presence of harmful substances can be minimized, thereby ensuring the safety of alternative medicines for use [125,126].

## 9. Conclusions and Scope for Future Research

Traditional medicines have been used for the past several decades to manage and support individuals with cancer even though they are not substitutes for modern cancer treatments such as surgery, chemotherapy, or radiation. They are used as complementary therapy to help alleviate symptoms, improve quality of life, and support immune function. Most of the traditional systems of medicines focus on the holistic approach of treatment towards cancer care, including herbal formulations, diet management, and lifestyle modification. Quality of life is a critical indicator of the health status of terminally ill cancer patients. It is vital to investigate the use of traditional medicines and their relevance to improving the quality of life of cancer patients. Therefore, the purpose of this study was to evaluate the use of complementary medicine for the management of cancer. By exploring the available alternative therapies, it is possible to address the challenges of current cancer treatment modalities in a more reliable and cost-effective manner. More scientific research is required in this area so that the concept of integrative oncology becomes more reliable and truthful. This review will serve as a foundation for future research in the area of integrative approaches to cancer care.

## Figures and Tables

**Table 1:** Important anticancer medicinal plants/formulations used in various traditional systems of medicine.

Medicinal Plant/ Formulation	Traditional System of Medicine	Major Active Compounds/ Ingredient Drugs	Type of Cancer	References
*Andrographis paniculata*	Ayurveda	Andrographolide	Leukemia, breast cancer, colon cancer	[47,48,49]
*Boswellia serrata*	Ayurveda	Triterpenic acids	Brain tumors	[50]
*Garcinia hanburyi* Hook.f.	TCM	Gambogic acid	Glioblastoma, breast cancer, lung, and liver	[51,52,53]
*Curcuma longa*	Ayurveda/TCM	Curcumin	Leukemia, lymphoma, melanoma colon, and gastric cancers	[54,55,56]
*Scutellaria baicalensis*	TCM	Wogonin	Cervical carcinoma	[57]
*Silybum marianum*	TCM	Silibinin	Prostate, colon, bladder, and lung	[58]
*Artemisia annua*	TCM	Artemisinin and its derivatives	Colon, ovarian, prostate	[59,60]
Rikkunshito	Kampo medicine	Hesperidin, isoliquiritigenin, atractylodin, glycycoumarin	Chemotherapy-induced dyspepsia, cancer cachexia	[61,62]
H9	Korean medicine	Psoraleae semen, evodia fruit, fennel, nutmeg, ginseng, alpiniae officinarum rhizome, sparganium rhizome, curcuma root, and cinnamon bark	Breast cancer	[63]
Juzentaihoto	Kampo	Astragalus root, cinnamon bark, rehmannia root, peony root, cnidium rhizome, atractylodes lancea rhizome, angelica root, ginseng, poria sclerotium, glycyrrhiza	Pancreatic cancer	[64]
*Tinospora cordifolia*	Ayurveda	20β-hydroxyecdysterone, cordioside, columbin	Ascites carcinoma	[65]
*Withania somnifera*	Ayurveda	Withanolides, withaferin	Colon, mammary, lung, prostate, skin, blood, liver, and kidney	[66]
*Phyllanthus amarus*	Ayurveda	Phyllanthin, niranthrin, phyltetralin, nirtetralin	Lung, cervical cancer	[67]
*Cynodon dactylon*	Ayurveda	Delphinidin-3-*O*-monoglucoside, cyanidin-3-*O*-monoglucoside	Lung, breast cancer	[68]
*Cyanthillium cinereum*	Ayurveda	Luteolin 7-*O*-glucuronide, Kaempferol 3-*O*-(6-*O*-acetyl) glycoside, apigenein-6-C-pentosyl-8-C-hexoside	Lung, breast cancer	[69]
*Euphorbia thymifolia*	Ayurveda	*p*-Coumaric acid, ferulic acid, kaempferol-3-glucuronide	Ascites carcinoma	[70]

TCM: Traditional Chinese Medicine.

## Data Availability

No new data were created or analyzed in this study. Data sharing is not applicable to this article.

## References

[1] Atanasov A.G., Zotchev S.B., Dirsch V.M., Supuran C.T. (2021). Natural products in drug discovery: Advances and opportunities. Nat. Rev. Drug Discov..

[2] Lee K.-H., Xiao Z., Cragg G.M., Kingston D.G.I., Newman D.J. (2005). Podophyllotoxins and analogs. Anticancer Agents from Natural Products.

[3] Siegel R.L., Miller K.D., Fuchs H.E., Jemal A. (2022). Cancer statistics. CA Cancer J. Clin..

[4] Ando K., Hu Q., Kasagi Y., Oki E., Mori M. (2021). Recent developments in cancer research: Expectations for a new remedy. Ann. Gastroenterol. Surg..

[5] Abdalla Y.O.A., Subramaniam B., Nyamathulla S., Shamsuddin N., Arshad N.M., Mun K.S., Awang K., Nagoor N.H. (2022). Natural Products for Cancer Therapy: A Review of Their Mechanism of Actions and Toxicity in the Past Decade. J. Trop. Med..

[6] Huang M., Lu J.-J., Ding J. (2021). Natural Products in Cancer Therapy: Past, Present and Future. Nat. Prod. Bioprospect..

[7] Van Vuuren S., Motlhatlego K., Netshia V. (2022). Traditionally used polyherbals in a southern African therapeutic context. J. Ethnopharmacol..

[8] Zimmermann-Klemd A.M., Reinhardt J.K., Winker M., Gründemann C. (2022). Phytotherapy in Integrative Oncology—An Update of Promising Treatment Options. Molecules.

[9] Sulaiman C., George B.P., Balachandran I., Abrahamse H. (2022). Photoactive Herbal Compounds: A Green Approach to Photodynamic Therapy. Molecules.

[10] Scharman E.J. (2005). Reserpine. Encyclopedia of Toxicology.

[11] Sulaiman C.T., Jyothi C.K., Unnithan J.K., Prabhukumar K.M., Balachandran I. (2020). Identification of suitable substitute for Sarpagandha (*Rauvolfia serpentina* (L.) Benth. ex Kurz) by phytochemical and pharmacological evaluation. Beni-Suef Univ. J. Basic Appl. Sci..

[12] Boeing H., Bechthold A., Bub A., Ellinger S., Haller D., Kroke A., Leschik-Bonnet E., Müller M.J., Oberritter H., Schulze M. (2012). Critical review: Vegetables and fruit in the prevention of chronic diseases. Eur. J. Nutr..

[13] Iqbal J., Abbasi B.A., Mahmood T., Kanwal S., Ali B., Shah S.A., Khalil A.T. (2017). Plant-derived anti-cancer agents: A green anticancer approach. Asian Pac. J. Trop. Biomed..

[14] Wang J., He C.Z., Dang C.L., Huang R.F. (2011). Genetic diversity and relationship of *Allium tchongchanense* and *A. wallichii* based on AFLP analysis. Guihaia.

[15] Bhandari J., Muhammad B., Thapa P., Shrestha B.G. (2017). Shrestha Study of phytochemical, anti-microbial, anti-oxidant, and anti-cancer properties of *Allium wallichii*. BMC Complement. Altern. Med..

[16] Sulaiman C.T., Shahida V., Balachandran I. (2015). Effect of Extraction Solvent on the Phytoconstituents of *Aegle marmelos* (L.) Correa. J. Nat. Remedies.

[17] Chockalingam V., Kadali S.S., Gnanasambantham P. (2012). Antiproliferative and antioxidant activity of *Aegle marmelos* (Linn.) leaves in Dalton’s Lymphoma Ascites transplanted mice. Indian J. Pharmacol..

[18] Akhouri V., Kumari M., Kumar A. (2020). Therapeutic effect of *Aegle marmelos* fruit extract against DMBA induced breast cancer in rats. Sci. Rep..

[19] Moongkarndi P., Kosem N., Luanratana O., Jongsomboonkusol S., Pongpan N. (2004). Antiproliferative activity of Thai medicinal plant extracts on human breast adenocarcinoma cell line. Fitoterapia.

[20] Baliga M.S. (2012). Review of the phytochemical, pharmacological and toxicological properties of *Alstonia Scholaris* Linn. R. Br (Saptaparna). Chin. J. Integr. Med..

[21] Xiang Y., Guo Z., Zhu P., Chen J., Huang Y. (2019). Traditional Chinese medicine as a cancer treatment: Modern perspectives of ancient but advanced science. Cancer Med..

[22] Li S., Chen X., Shi H., Yi M., Xiong B., Li T. (2025). Tailoring traditional Chinese medicine in cancer therapy. Mol. Cancer.

[23] Lin F., Zhang G., Yang X., Wang M., Wang R., Wan M., Wang J., Wu B., Yan T., Jia Y. (2023). A network pharmacology approach and experimental validation to investigate the anticancer mechanism and potential active targets of ethanol extract of Wei-Tong-Xin against colorectal cancer through induction of apoptosis via PI3K/AKT signaling pathway. J. Ethnopharmacol..

[24] Liu J., Wang S., Zhang Y., Fan H., Lin H. (2015). Traditional Chinese medicine and cancer: History, present situation, and development. Thorac. Cancer.

[25] Hu B., An H.M., Shen K.P. (2009). Documents research of TCM cancer treatment of SCI journals. Chin. J. Ethnomed. Ethnopharm..

[26] Hou W., Liu J., Shi W.G., Lin H.S. (2013). Multi-center, randomized, controlled clinical study of radiation pneumonitis treated with compound matrine injection in primary lung cancer patients. Chin. J. New Drugs.

[27] Lin H., Sun G., Qin F., Cao Y., Wang X., Chen J., Wang X., Huang H. (2013). A randomized, double-blinded, drug-controlled and multicentre clinical trial of chemotherapy assisted with Jinlong capsule on gastric cancer. Cancer Res. Prev. Treat..

[28] Mukherjee P.K., Nema N.K., Venkatesh P., Debnath P.K. (2012). Changing scenario for promotion and development of Ayurveda—Way forward. J. Ethnopharmacol..

[29] Jaiswal Y.S., Williams L.L. (2016). A glimpse of Ayurveda—The forgotten history and principles of Indian traditional medicine. J. Tradit. Complement. Med..

[30] Bhishagratha K.L. (1991). Sushruta Samhita.

[31] Balachandran P., Govindarajan R. (2005). Cancer: An ayurvedic perspective. Pharmacol. Res..

[32] Hirsch H.A., Iliopoulos D., Joshi A., Zhang Y., Jaeger S.A., Bulyk M., Tsichlis P.N., Liu X.S., Struhl K. (2010). A transcriptional signature and common gene networks link cancer with lipid metabolism and diverse human diseases. Cancer Cell.

[33] Chen Y., Zhu J., Lum P.Y., Yang X., Pinto S., MacNeil D.J., Zhang C., Lamb J., Edwards S., Sieberts S.K. (2008). Variations in DNA elucidate molecular networks that cause disease. Nature.

[34] Hanahan D., Weinberg R.A. (2011). Hallmarks of cancer: The next generation. Cell.

[35] Sumantran V.N., Tillu G. (2012). Cancer, Inflammation, and Insights from Ayurveda. Evid.-Based Complement. Altern. Med..

[36] Sharma H., Wallace R.K. (2020). Ayurveda and Epigenetics. Medicina.

[37] Mondal P., Natesh J., Penta D., Meeran S.M. (2022). Progress and Promises of Epigenetic Drugs and Epigenetic Diets in Cancer Prevention and Therapy: A clinical update. Semin. Cancer Biol..

[38] Meeran S.M., Patel S.N., Li Y., Shukla S., Tollefsbol T.O. (2012). Bioactive dietary supplements reactivate ER expression in ER-negative breast cancer cells by active chromatin modifications. PLoS ONE.

[39] Shim E.B., Lee S., Kim J.Y., Earm Y.E. (2008). Physiome and sasang constitutional medicine. J. Physiol. Sci..

[40] Kumar H., Song S.-Y., More S.V., Kang S.-M., Kim B.-W., Kim I.-S., Choi D.-K. (2013). Traditional Korean East Asian medicines and herbal formulations for cognitive impairment. Molecules.

[41] Yoon S.W., Jeong J.S., Kim J.H., Aggarwal B.B. (2013). ancer Prevention and Therapy: Integrating Traditional Korean Medicine Into Modern Cancer Care. Integr. Cancer Ther..

[42] Motoo Y., Seki T., Tsutani K. (2011). Traditional Japanese medicine, Kampo: Its history and current status. Chin. J. Integr. Med..

[43] Bashir F., Akhtar J., Anjum N., Alam S., Khan A.A. (2020). Concept of Sartān (Cancer) and Anti-cancerous drugs in Unani System of Medicine. Int. J. Curr. Sci. Multidiscip. Res..

[44] Mustehasan, Naushin S., Alam M. (2019). Role of Diet in the Prevention and Management of Cancer (Saraöän) in Unani Medicine. Hippocrat. J. Unani Med..

[45] Zohar A.M.A.M.I. (1986). Kitabul Taiseer, (Urdu Translation by CCRUM).

[46] Sowmyalakshmi S., Nur-e-Alam M., Akbarsha M.A., Thirugnanam S., Rohr J., Chendil D. (2005). Investigation on *Semecarpus Lehyam*—A Siddha medicine for breast cancer. Planta.

[47] Kumar R.A., Sridevi K., Kumar N.V., Nanduri S., Rajagopal S. (2004). Anticancer and immunostimulatory compounds from *Andrographis paniculata*. J. Ethnopharmacol..

[48] Jada S.R., Subur G.S., Matthews C., Hamzah A.S., Lajis N.H., Saad M.S., Stevens M.F., Stanslas J. (2007). Semisynthesis and in vitro anticancer activities of andrographolide analogues. J. Phytochem..

[49] Matsuda T., Kuroyanagi M., Sugiyama S., Umehara K., Ueno A., Nishi K. (1994). Cell differentiation-inducing diterpenes from *Andrographis paniculata* Nees. Chem. Pharm. Bull..

[50] Streffer J.R., Schabet M., Dichgans J., Weller M. (2001). Response of radiochemotherapy-associated cerebral edema to a phytotherapeutic agent, H15. Neurology.

[51] Panthong A., Norkaew P., Kanjanapothi D., Taesotikul T., Anantachoke N., Reutrakul V. (2007). Anti-inflammatory, analgesic and antipyretic activities of the extract of gamboge *from Garcinia hanburyi* Hook f. J. Ethnopharmacol..

[52] Wu Z.-Q., Guo Q.-L., You Q.-D., Zhao L., Gu H.-Y. (2004). Gambogic acid inhibits proliferation of human lung carcinoma SPC-A1 cells in vivo and in vitro and represses telomerase activity and telomerase reverse transcriptase mRNA expression in the cells. Biol. Pharm. Bull..

[53] Qi Q., Gu H., Yang Y., Lu N., Zhao J., Liu W., Ling H., You Q.-D., Wang X., Guo Q. (2008). Involvement of matrix metalloproteinase 2 and 9 in gambogic acid induced suppression of MDA-MB-435 human breast carcinoma cell lung metastasis. J. Mol. Med..

[54] Goel A., Kunnumakkara A.B., Aggarwal B.B. (2008). Curcumin as “Curecumin”: From kitchen to clinic. Biochem. Pharmacol..

[55] López-Lázaro M. (2008). Anticancer and carcinogenic properties of curcumin: Considerations for its clinical development as a cancer chemopreventive and chemotherapeutic agent. Mol. Nutr. Food Res..

[56] Rajamanickam S., Velmurugan B., Kaur M., Singh R.P., Agarwal R. (2010). Chemoprevention of intestinal tumorigenesis in APC^min/+^ mice by silibinin. Cancer Res..

[57] Yang L., Zhang H.-W., Hu R., Yang Y., Qi Q., Lu N., Liu W., Chu Y.-Y., You Q.-D., Guo Q.-L. (2009). Wogonin induces G_1_ phase arrest through inhibiting Cdk4 and cyclin D1 concomitant with an elevation in p21^Cip1^ in human cervical carcinoma HeLa cells. Biochem. Cell Biol..

[58] Singh R.P., Raina K., Deep G., Chan D., Agarwal R. (2009). Silibinin suppresses growth of human prostate carcinoma PC-3 orthotopic xenograft via activation of extracellular signal-regulated kinase 1/2 and inhibition of signal transducers and activators of transcription signaling. Clin. Cancer Res..

[59] Jiao Y., Ge C.-M., Meng Q.-H., Cao J.-P., Tong J., Fan S.-J. (2007). Dihydroartemisinin is an inhibitor of ovarian cancer cell growth. Acta Pharmacol. Sin..

[60] He Q., Shi J., Shen X.-L., An J., Sun H., Wang L., Hu Y.-J., Sun Q., Fu L.-C., Sheikh M.S. (2010). Dihydroartemisinin upregulates death receptor 5 expression and cooperates with TRAIL to induce apoptosis in human prostate cancer cells. Cancer Biol. Ther..

[61] Mogami S., Hattori T. (2014). Beneficial effects of rikkunshito, a Japanese kampo medicine, on gastrointestinal dysfunction and anorexia in combination with Western drug: A systematic review. Evid.-Based Complement. Altern. Med..

[62] Motoo Y., Cameron S. (2022). Kampo medicines for supportive care of patients with cancer: A brief review. Integr. Med. Res..

[63] Lee J., Son Y.H., Kwon Y., Park S.Y., Koo B., Jung S.H. (2017). Anticancer Effects of a Korean Herbal Medicine Formula (H9) via AMPK and HER2-PI3K/Akt Signaling in Breast Cancer Cells. Phytother. Res..

[64] Shimizu M., Takayama S., Kikuchi A., Arita R., Ono R., Ishizawa K., Ishii T. (2021). Kampo Medicine Treatment for Advanced Pancreatic Cancer: A Case Series. Front. Nutr..

[65] Jagetia G.C., Rao S.K. (2006). Evaluation of the antineoplastic activity of guduchi (*Tinospora cordifolia*) in Ehrlich ascites carcinoma bearing mice. Biol. Pharm. Bull..

[66] Singh N., Yadav S., Rao A.S., Nandal A., Kumar S., Ganaie S., Narasihman B. (2021). Review on anticancerous therapeutic potential of *Withania somnifera* (L.) Dunal. J. Ethnopharmacol..

[67] Paul S., Patra D., Kundu R. (2019). Lignan enriched fraction (LRF) of *Phyllanthus amarus* promotes apoptotic cell death in human cervical cancer cells in vitro. Sci. Rep..

[68] Sulaiman C.T., Ramesh P.R., Mahesh K., Anandan E.M., Praveen M., Balachandran I. (2022). Metabolite profiling of *Cyanthillium cinereum* (L.) H. Rob. and its herbal formulation by tandem mass spectroscopic analysis. Nat. Prod. Res..

[69] Sulaiman C., Deepak M., Praveen T., Lijini K., Salman M., Sadheeshnakumari S., Balachandran I. (2022). Metabolite profiling and anti-cancer activity of two medicinally important *Euphorbia* species. Med. Omics.

[70] Mugomeri E., Chatanga P., Chakane N. (2016). Medicinal herbs used by HIV positive people in Lesotho. Afr. J. Tradit. Complement. Altern. Med..

[71] Malangu N. (2007). Self-reported use of traditional, complementary and over-the-counter medicines by HIV-infected patients on antiretroviral therapy in Pretoria, South Africa. Afr. J. Tradit. Complement. Altern. Med..

[72] Langlois-Klassen D., Kipp W., Jhangri G.S., Rubaale T. (2007). Use of traditional herbal medicine by AIDS patients in Kabarole District, western Uganda. Am. J. Trop. Med. Hyg..

[73] Illamola S.M., Amaeze O.U., Krepkova L.V., Birnbaum A.K., Karanam A., Job K.M., Bortnikova V.V., Sherwin C.M., Enioutina E.Y. (2020). Use of herbal medicine by pregnant women: What physicians need to know. Front. Pharmacol..

[74] Jain S., Dwivedi J., Jain P.K., Satpathy S., Patra A. (2016). Medicinal Plants for Treatment of Cancer: A Brief Review. Pharmacogn. J..

[75] Sasidharan S., Saravanan D., Chen Y., Sundram K.M., Latha L.Y. (2011). Extraction, isolation and characterization of bioactive compounds from plants’ extracts. Afr. J. Tradit. Complement. Altern. Med..

[76] Lakshmi Priya M., Bhanu Priya K., Kotakadi V.S., Josthna P. (2015). Herbal and Medicinal Plants Molecules Towards Treatment of Cancer: A Mini Review. Am. J. Ethnomedicine.

[77] Umadevi M., Sampath Kumar K.P., Bhowmik D., Duraivel S. (2013). Traditionally Used Anticancer Herbs in India. J. Med. Plants Stud..

[78] Gordon M.C., David J. (2005). Plants as a source of anticancer agents. J. Ethnopharmacol..

[79] Kuo Y.-T., Chang T.-T., Muo C.-H., Wu M.Y., Sun M.F., Yeh C.C., Yen H.R. (2017). Use of complementary traditional Chinese medicines by adult cancer patients in Taiwan: A nationwide population-based study. Integr. Cancer Ther..

[80] Jang J.Y., Kim D., Im E., Kim N.D. (2025). Etoposide as a Key Therapeutic Agent in Lung Cancer: Mechanisms, Efficacy, and Emerging Strategies. Int. J. Mol. Sci..

[81] Jermini M., Dubois J., Rodondi P.-Y., Zaman K., Buclin T., Csajka C., Orcurto A., Rothuizen L.E. (2019). Complementary medicine use during cancer treatment and potential herb-drug interactions from a cross-sectional study in an academic centre. Sci. Rep..

[82] Dhanoa A., Yong T.L., Yeap S.J.L., Lee I.S.Z., Singh V.A. (2014). Complementary and alternative medicine use amongst Malaysian orthopaedic oncology patients. BMC Complement. Altern. Med..

[83] Liu T.G., Xiong S.Q., Yan Y., Zhu H., Yi C. (2012). Use of Chinese herb medicine in cancer patients: A survey in southwestern China. Evid.-Based Complement. Altern. Med..

[84] Karadeniz C., Pinarli F.G., Oguz A., Gursel T., Canter B. (2007). Complementary/alternative medicine use in a pediatric oncology unit in Turkey. Pediatr. Blood Cancer.

[85] Nazik E., Nazik H., Api M., Kale A., Aksu M. (2012). Complementary and alternative medicine use by gynecologic oncology patients in Turkey. Asian Pac. J. Cancer Prev..

[86] Molassiotis A., Fernandez-Ortega P., Pud D., Ozden G., Platin N., Hummerston S., Scott J.A., Panteli V., Gudmundsdottir G., Selvekerova S. (2005). Complementary and alternative medicine use in colorectal cancer patients in seven European countries. Complement. Ther. Med..

[87] Tarhan O., Muslu U., Somali I., Erten C., Alacacioglu A., Varol S., Aslan L. (2009). An analysis of the use of complementary and alternative therapies in patients with breast cancer. Breast Care.

[88] Ezeome E.R., Anarado A.N. (2007). Use of complementary and alternative medicine by cancer patients at the University of Nigeria Teaching Hospital, Enugu, Nigeria. BMC Complement. Altern. Med..

[89] Naja F., Anouti B., Shatila H., Akel R., Haibe Y., Tfayli A. (2017). Prevalence and correlates of complementary and alternative medicine use among patients with lung cancer: A cross-sectional study in Beirut, Lebanon. Evid.-Based Complement. Altern. Med..

[90] Puataweepong P., Sutheechet N., Ratanamongkol P. (2012). A survey of complementary and alternative medicine use in cancer patients treated with radiotherapy in Thailand. Evid.-Based Complement. Altern. Med..

[91] Wong L.C., Chan E., Tay S., Lee K.M., Back M. (2010). Complementary and alternative medicine practices among Asian radiotherapy patients. Asia-Pac. J. Clin. Oncol..

[92] Yin S.-Y., Wei W.-C., Jian F.-Y., Yang N.-S. (2013). Therapeutic Applications of Herbal Medicines for Cancer Patients. Evid.-Based Complement. Altern. Med..

[93] Chen X., Hu Z.-P., Yang X.-X., Huang M., Gao Y., Tang W., Chan S.Y., Dai X., Ye J., Ho P.C.-L. (2006). Monitoring of immune responses to a herbal immuno-modulator in patients with advanced colorectal cancer. Int. Immunopharmacol..

[94] Lam M. (2003). Natural medicine. Beating Cancer with Natural Medicine.

[95] Rees R.W., Feigel I., Vickers A., Zollman C., Mc Gurk R., Smith C. (2000). Prevalence of complementary therapy use by women with breast cancer: A population based survey. Eur. J. Cancer..

[96] Gray R.E., Fitch M., Goel V., Franssen E., Labrecque M. (2003). Utilization of complementary/alternative services by women with breast cancer. J Health Soc Policy.

[97] Henderson J.W., Donattele R.J. (2004). Complementary and alternative medicine use by women after completion of allopatice treatment for breast cancer. Altern. Ther. Health Med..

[98] Morris K.T., Johnson N., Homer L., Walts D. (2000). A comparison of complementary therapy use between breast cancer patients and with other primary tumor sites. Am. J. Surg..

[99] Jentzsch V., Davis J., Djamgoz M.B.A. (2020). Pancreatic Cancer (PDAC): Introduction of Evidence-Based Complementary Measures into Integrative Clinical Management. Cancers.

[100] Djamgoz M.B.A., Jentzsch V. (2022). Integrative Management of Pancreatic Cancer (PDAC): Emerging Complementary Agents and Modalities. Nutr. Cancer.

[101] Ramamoorthy A. (2015). Integrative oncology in Indian subcontinent: An overview. J. Clin. Diagn. Res..

[102] Mulabagal V., Subbaraju G.V., Rao C.V., Sivaramakrishna C., Dewitt D.L., Holmes D., Sung B., Aggarwal B.B., Tsay H.-S., Nair M.G. (2009). Withanolide sulfoxide from Aswagandha roots inhibits nuclear transcription factor-kappa-B, cyclooxygenase and tumor cell proliferation. Phytother Res..

[103] Riyasdeen A., Periasamy V.S., Paul P., Alshatwi A.A., Akbarsha M.A. (2012). Chloroform Extract of Rasagenthi Mezhugu, a Siddha Formulation, as an Evidence-Based Complementary and Alternative Medicine for HPV-Positive Cervical Cancers. Evid. Based Complement. Altern. Med..

[104] Rocha V., Ladas E.J., Lin M., Cacciavillano W., Ginn E., Kelly K.M., Chantada G., Castillo L. (2017). Beliefs and determinants of use of traditional complementary/alternative medicine in pediatric patients who undergo treatment for cancer in South America. J. Glob. Oncol..

[105] Samano E.S.T., Goldenstein P.T., Ribeiro L.d.M., Lewin F., Filho E.S.V., Soares H.P., del Giglio A. (2004). Praying correlates with higher quality of life: Results from a survey on complementary/alternative medicine use among a group of Brazilian cancer patients. Sao Paulo Med. J..

[106] Chen Z., Gu K., Zheng Y., Zheng W., Lu W., Shu X.O. (2008). The use of complementary and alternative medicine among Chinese women with breast cancer. J. Altern. Complement. Med..

[107] McQuade J.L., Meng Z., Chen Z., Wei Q., Zhang Y., Bei W., Palmer J.L., Cohen L. (2012). Utilization of and attitudes towards traditional Chinese medicine therapies in a Chinese cancer hospital: A survey of patients and physicians. Evid.-Based Complement. Altern. Med..

[108] Chen G., Qiao T.-T., Ding H., Li C.-X., Zheng H.-L., Chen X.-L., Hu S.-M., Yu S.-Y. (2015). Use of Chinese herbal medicine therapies in comprehensive hospitals in central China: A parallel survey in cancer patients and clinicians. J. Huazhong Univ. Sci. Technol. [Med. Sci.].

[109] Aggarwal B.B., Ichikawa H., Garodia P., Weerasinghe P., Sethi G., Bhatt I.D., Pandey M.K., Shishodia S., Nair M.G. (2006). From traditional Ayurvedic medicine to modern medicine: Identification of therapeutic targets for suppression of inflammation and cancer. Expert Opin. Ther. Targets.

[110] Coopoosamy R.M. (2012). An ethnobotanical study of medicinal plants used by traditional healers in Durban, South Africa. Afr. J. Pharm. Pharmacol..

[111] Koduru S., Grierson D.S., Afolayan A.J. (2007). Ethnobotanical information of medicinal plants used for treatment of cancer in the Eastern Cape Province, South Africa. Curr. Sci..

[112] Thring T., Weitz F. (2006). Medicinal plant use in the bredasdorp/elim region of the southern overberg in the Western Cape Province of South Africa. J. Ethnopharmacol..

[113] Semenya S., Maroyi A., Potgieter M., Erasmus L. (2013). Herbal medicines used by Bapedi traditional healers to treat reproductive ailments in the Limpopo Province, South Africa. Afr. J. Tradit. Complement. Altern. Med..

[114] Twilley D., Rademan S., Lall N. (2020). A review on traditionally used South African medicinal plants, their secondary metabolites and their potential development into anticancer agents. J. Ethnopharmacol..

[115] Sagbo I.J., Otang-Mbeng W. (2021). Plants Used for the Traditional Management of Cancer in the Eastern Cape Province of South Africa: A Review of Ethnobotanical Surveys, Ethnopharmacological Studies and Active Phytochemicals. Molecules.

[116] Ravipati A.S., Zhang L., Koyyalamudi S.R., Jeong S.C., Reddy N., Bartlett J., Smith P.T., Shanmugam K., Münch G., Wu M.J. (2012). Antioxidant and anti-inflammatory activities of selected Chinese medicinal plants and their relation with antioxidant content. BMC Complement. Altern. Med..

[117] Banerjee S., Nau S., Hochwald S.N., Xie H., Zhang J. (2022). Anticancer properties and mechanisms of botanical derivatives. Phytomed. Plus.

[118] Joshi P., Yadaw G., Joshi S., Semwal R., Semwal D. (2020). Antioxidant and anti-inflammatory activities of selected medicinal herbs and their polyherbal formulation. S. Afr. J. Bot..

[119] Pan M.-H., Chiou Y.-S., Tsai M.-L., Ho C.-T. (2011). Anti-inflammatory activity of traditional Chinese medicinal herbs. J. Tradit. Complement. Med..

[120] Hodzic Z., Pasalic H., Memisevic A., Srabovic M., Saletovic M., Poljakovic M. (2009). The influence of total phenols content on antioxidant capacity in the whole grain extract. Eur. J. Sci. Res..

[121] Nam S.-Y., Kim K.-Y., Kim M.H., Jang J.-B., Rah S.-Y., Lee J.-M., Kim H.-M., Jeong H.-J. (2017). Anti-inflammatory effects of a traditional Korean medicine: Ojayeonjonghwan. Pharm. Biol..

[122] Sunagawa M., Yamaguchi K., Tsukada M., Ebihara N., Ikemoto H., Hisamitsu T. (2018). Kampo (Traditional Japanese Herbal) Formulae for Treatment of Stomatitis and Oral Mucositis. Medicines.

[123] Hecht F., Zocchi M., Alimohammadi F., Harris I.S. (2024). Regulation of antioxidants in cancer. Mol. Cell.

[124] Zappavigna S., Cossu A.M., Grimaldi A., Bocchetti M., Ferraro G.A., Nicoletti G.F., Filosa R., Caraglia M. (2020). Anti-Inflammatory Drugs as Anticancer Agents. Int. J. Mol. Sci..

[125] Vlietinck A., Pieters L., Apers S. (2009). Legal requirements for the quality of herbal substances and herbal preparations for the manufacturing of herbal medicinal products in the European union. Planta Medica.

[126] Chen Y., Wang L., Liu Q., Yang S., Wang C. (2023). Advancing herbal medicine: Enhancing product quality and safety through robust quality control practices. Front. Pharmacol..

